# Optimization of Graphene Nanoplatelets Dispersion and Its Performance in Cement Mortars

**DOI:** 10.3390/ma15207308

**Published:** 2022-10-19

**Authors:** Yong Zhou, Yuliang Wang, Tianming Gao, Yifeng Ling, Nengdong Jiang, Abdullah M. Tawfek, Huaqiang Yuan

**Affiliations:** 1 Shandong Hi-Speed Group Co., Ltd., Jinan 250002, China; 2 School of Qilu Transportation, Shandong University, Jinan 250002, China; 3 Bridge and Tunnel Engineering, Sana’s University, Sanaa 12544, Yemen

**Keywords:** graphene nanoplatelets, dispersion, surfactant, cement mortar

## Abstract

As promising next-generation conducting materials, Graphene Nanoplatelets (GNPs) have been widely used to enhance the mechanical and pressure-sensitive properties of cement-based materials. However, this beneficial effect highly depended on its dispersion. In this study, polyvinyl pyrrolidone (PVP) surfactant, high-speed shear, and ultrasonication were used to disperse GNPs. To fully exert the mechanical and pressure-sensitive properties and enhance the dispersion effect of GNPs in cement-based materials, the dispersing method parameters, including PVP concentration, ultrasonication time, shear time, and rate, were optimized. The dispersion degree of GNPs was evaluated by absorbance. The results show that the optimal dispersion parameters were 10 mg/mL of PVP concentration, 15 min of ultrasonication time, 15 min of shear time, and 8000 revolutions per minute (rpm) of shear rate. In addition, the effect of GNPs dosage (0.05, 0.1, 0.3, 0.5, 0.7, and 1.0 wt%) on the setting time, flowability, and mechanical and pressure-sensitive properties of cement mortar were examined. Results reveal that the optimum dosage of GNPs was found at 1.0 wt%.

## 1. Introduction

Cement concrete is widely used in civil buildings and inevitably faces various long-term loading effects and the erosion of the harsh environment, which will cause structural cracks, even resulting in the collapse of the construction building. Much effort has been made to regularly monitor the health of concrete structures, aiming at reducing such risks [1]. The conventional approaches are to embed piezoelectric sensors and strain gages in concrete structures [2,3]. Nevertheless, these sensors and strain gages inherently have poor durability and stability in defective electrical conductivity and usually require expensive external facilities [4,5]. Recently, many researchers have reported that pressure-sensitive function can be achieved by adding conductive filler in cement-based materials, which provides an alternative to monitoring the health of concrete structures [6,7].

Nanomaterials like carbon nanotubes (CNTs) [8,9], nanocarbon fiber (NCF) [10,11], and GNPs are common conductive fillers in cement-based materials [12]. Compared with CNTs and NCT, GNPs have higher solubility in aqueous solution because of the abundant hydrophilic functional groups, including hydroxyl, carboxyl, and carbonyl functional groups on the basal plane of graphene [13,14,15]. Moreover, GNPs have a higher surface area and a wrinkled morphology, which increases the nucleation sites and roughness of the interface between GNPs and the cement-based materials [13,16,17,18]. In previous studies, Sun et al. [19] investigated the electrically conductive and pressure-sensitive behaviors of cementitious composites filled with 0–10 wt% of GNPs under mechanical loading and suggested that the GNP-modified composites can be considered as stress sensors for health monitoring. Xu et al. [20] found that the addition of GNPs would lead to a notable decrease in electrical resistivity and an excellent pressure sensitivity in cement-based materials. 

At present, the most common methods to disperse the GNPs are adding a surfactant, high-speed shear, and ultrasonication [21,22,23]. The shear force produced from high-speed shear can separate the weakly coupled GNPs into graphene [24]. Ultrasonication disperses agglomerated GNPs with bubbles generated by strong local shear force, and the GNPs aggregations are scattered by bubble forming and rupturing [13]. However, GNPs will tend to re-agglomerate over time due to the gain in entropy after the treatment of high-speed shear or ultrasonication [25,26]. Therefore, a surfactant has been frequently used to prevent such re-agglomeration since it can adsorb on the surface of GNPs particles to cause steric hindrance among them [23,27,28,29]. Wei, et al. [30] prepared the GNPs suspension using seven surfactants and found that PVP had the optimal dispersion effect in the aqueous solution. Some researchers first ultrasonicated and then added surfactant into GNPs′ aqueous solution, which finally resulted in a relatively satisfactory dispersion effect [31,32]. In addition, some studies have used ultrasonication to prepare GNPs suspensions. Still, prolonged ultrasonication treatment time can damage the size and shape of graphene, which in turn affects its pressure sensitivity [33,34]. In order to prepare graphene suspensions with good pressure sensitivity properties, high-speed shear was used to replace part of the ultrasonication [35]. Thus, it would be effective in the dispersion of GNPs to combine these three methods. Nevertheless, with numerous parameters involved, such as PVP concentration, ultrasonication time, high-speed shear time, and rate, it is key to optimize these parameters to secure a balance between energy consumption and dispersion effect. However, limited research has been reported on the optimization parameters of the combined dispersion methods.

Thus, in this study, a combined dispersion method (PVP surfactant, high-speed shear, and ultrasonication) were used to disperse GNPs in cement mortar. Its parameters, including PVP concentration, ultrasonication time, shear time, and rate, were optimized by investigating their effects on dispersion degree. Additionally, the effect of GNPs dosage on the setting time, flowability, and mechanical and pressure-sensitive properties of cement mortar was also addressed using the optimal dispersion method.

## 2. Materials and Methods

### 2.1. Raw Materials 

P.O. 42.5 Ordinary Portland cement [36] and Class-II fly ash [37] were used in this study, and their chemical compositions were given in Table 1 based on the X-ray fluorescence (XRF) test. GNPs were purchased from XG Science Co. Ltd., and their properties are listed in Table 2.

The quartz sand had a grain diameter from 124 to 178 μm. In order to maintain workability, the superplasticizer was used. The viscosity of the mortar was modified by a Hypromellose thickener agent with a viscosity grade of 150. Non-ionic surfactant polyvinyl pyrrolidone (PVP) was purchased from Sinopharm Chemical Reagent Co., Ltd. and used for GNPs dispersion. Its parameters are presented in Table 3.

### 2.2. Optimization of Dispersion Parameters

**Figure 1:**
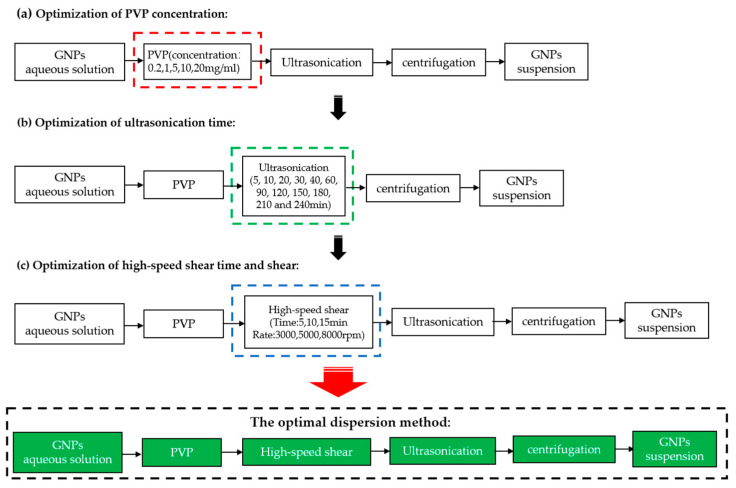
Flowchart of optimal dispersion parameter. (**a**) Optimization of PVP concentration; (**b**) Optimization of ultrasonication time; (**c**) Optimization of high-speed shear time and rate.

The GNPs suspension preparation flowchart is shown in Figure 1. Three steps (a, b, c) were taken to optimize PVP concentration, ultrasonication time, and high-speed shear time and rate.

(a) Optimization of PVP concentration: PVP of 0.02, 0.1, 0.5, 1, and 2% concentration (wt% of the GNPs) were mixed with GNPs (1 g) in 100 mL water and stirred evenly with a glass rod. For each PVP concentration, the number of prepared samples in one group is six. Three mixed GNPs suspensions were subjected to a 30 min ultrasonication, and the other three suspensions were ultrasonicated for 60 min at 650 W. Finally, the absorbance of GNPs suspension was measured after centrifugation at 8000 rpm for 15 min. In order to obtain a stable suspension as quickly as possible. Centrifugation is used to remove the slag in the GNPs suspension, and then take the upper layer solution to measure the absorbance. After the measurement, the solution was cured at a temperature of 20 ± 3 °C and humidity of 95 ± 5%. Then, the absorbance was tested again after curing for 1, 3, and 120 days and the dispersion stability was evaluated by calculating the rate of absorbance loss (R) at 3 and 120 days, respectively. The absorbance loss rate was calculated by Equation (1):
  (1) $$R=[(Ab_{1d}-Ab_{t})/Ab_{1d}]\times 100$$
  where R is the rate of absorbance loss; A*b*_1__*d*_ is the absorbance of GNPs suspension at 1*d*; A*b_t_* is the absorbance of GNPs suspension at 3 d or 120 d. The optimal PVP concentration was determined by the value of absorbance and the absorbance loss rate.
(b) Optimization of ultrasonication time: The GNPs suspension with optimal PVP concentration was used to optimize the ultrasonication time. The absorbance and the color of the GNPs suspension were evaluated on various ultrasonication time of 5, 10, 20, 30, 40, 60, 90, 120, 150, 180, 210, and 240 min. For each ultrasonication time, three mixed GNPs suspensions were tested. The optimal ultrasonication time was then determined.
(c) Optimization of high-speed shear time and rate to replace partial ultrasonication: The GNPs suspension with optimal PVP concentration and ultrasonication time was used to optimize the high-speed shear time and shear. The shear time of 5, 10, and 15 min, and the shear rate of 3000, 5000, and 8000 rpm were selected as variables. For each high-speed shear time and rate, three mixed GNPs suspensions were tested. After similar procedures asin (b), the optimal high-speed shear time and rate were determined by the value of absorbance. It should be noted that since the introduction of the shear treatment might shift the optimal ultrasonication time towards smaller values, thus a proper adjustment to the ultrasonication time was needed according to the experimental results. Finally, according to the above three steps, the optimal dispersion method was determined.

The dispersion effect of GNPs was characterized by absorbance, and the dispersion degree of GNPs in various solutions was evaluated using UV-vis spectroscopy (UV-8000 spectrophotometer, Yuanxi Instrument, Shanghai, China) with a wavelength of 268 nm. According to the Beer–Lambert Law, the absorbance can be measured as follow:

(2) A=Kbc

where *c* and b are the concentration and the path length through the absorbing samples, respectively. For each species and wavelength, K is a constant known as the molar absorptivity or extinction coefficient. After centrifugation, the GNPs suspension was diluted 100 times and measured in the wavelength of 268 nm. Over time, it is expected that the suspended GNPs particles will aggregate and settle down at the bottom. By measuring the change in the optical density of samples, the concentration of particles in the solution could be obtained over time.

### 2.3. Mix Proportion and Preparation of GNPs Cement Mortar

The mixed proportions of cement mortar are presented in Table 4. A total of seven groups of the GNPs dosage (0, 0.05, 0.1, 0.3, 0.5, 0.7, and 1.0 wt% of the cement material) were prepared, and a fixed w/c ratio of 0.264 was used for all mixtures. GNPs have a bigger specific surface, which leads to more water required to keep the flowability of mixtures. Therefore, thickener and superplasticizer were used to increase workability with the dosage of 0.1% and 0.35% by mass of cement, respectively. The GNPs were dispersed in the aqueous solution by the optimal dispersion method. The fly ash and cement paste were first mixed at 150 rpm for 2 min. Then, GNPs suspensions were mixed with the cement and quartz sand mixture at 400 rpm for 2 min to perform the flowability, flexural strength, compressive strength, and pressure-sensitive property tests. The setting time was tested on paste with the same mix proportions, excluding quartz sand.

### 2.4. Testing Methods

#### 2.4.1. Setting Time and Flowability

After mixing, the setting time of GNPs cement paste was measured following the procedure outlined in ASTM C 305. A mini slump test was performed to determine the flowability of the fresh cement mortar as described in China National Standard (GB/T 2419-2016). A conical mold with a base diameter of 60 mm, top diameter of 36 mm, and height of 60 mm was filled with fresh cement mortar and vertically pulled upwards. The mean value of two perpendicular spread diameters of the cement mortar was reported as the flowability.

#### 2.4.2. Mechanical Strength Test

Prisms with a size of 40 mm × 40 mm × 160 mm were cast, and subsequently, the samples were demolded after 24 h and cured in a standard curing room with a temperature of 20 ± 1 °C and relative humidity of 98 ± 2%. The flexural and compressive strengths were measured according to China National Standard GB/T 17,671 at 3, 7, and 28 days. Three specimens were tested for each mixture.

#### 2.4.3. Pressure-Sensitive Measurements

A four-probe method was used for the measurement of potential differences across the specimen to remove the effects of contact resistance. The details of the measuring procedure can be found in [38]. The mortar specimens of 100 × 100 × 100 mm with GNPs dosage of 0.05%, 0.1%, 0.3%, 0.5%, 0.7%, 1% by mass of binder were cast. All specimens were cured in a curing room for 28 days and dried at 105 °C in an oven for 1 day before testing. Four stainless steel meshes were embedded in the specimens so that the average electrical resistance could be measured across the entire cross-section to minimize the effects of spatial variability. The diameter and spacing of the steel mesh were chosen to be 1 mm and 4 mm, respectively. During the measurement, a direct current (DC) power was used. Voltage and current were monitored by a digital multimeter supplied by Keithley Instruments. A constant DC was applied to the outer two current probes while the potential difference was measured using the inner two voltage probes. The ohmic behavior of the material was investigated by monitoring its resistance over current. The specimen was then loaded under uniaxial compression by a universal testing machine. A cyclical load, i.e., 10–40 kN, with 40 cycles, was used for the loading process. Data from the last five cycles were used to calculate the average resistance change rate.

## 3. Results

### 3.1. Optimization of PVP Concentration

**Figure 2:**
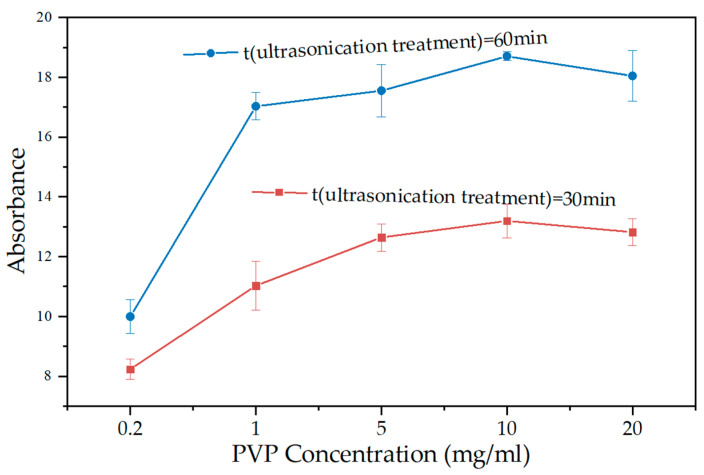
Absorbance of GNPs suspension at different PVP concentrations.

Figure 2 shows the dispersion effect of PVP at various concentrations. The absorbance of GNPs suspension steadily increased as the PVP concentrations increased to 10 mg/mL and then decreased for the PVP concentrations exceeding 10 mg/mL. These experimental results were similar to findings for other dispersed stabilized nanomaterials [28,38]. The reason for this phenomenon was that there was a limiting concentration (critical micelle concentration), beyond which the adsorption of PVP on GNPs reached saturation [39,40]. The hydrophobic groups of PVP tended to escape from the aqueous environment and form an inner core by self-polymerization inside the solution. In contrast, hydrophilic groups faced outward to form a shell in contact with water and formed a gel cluster [40,41].

**Figure 3:**
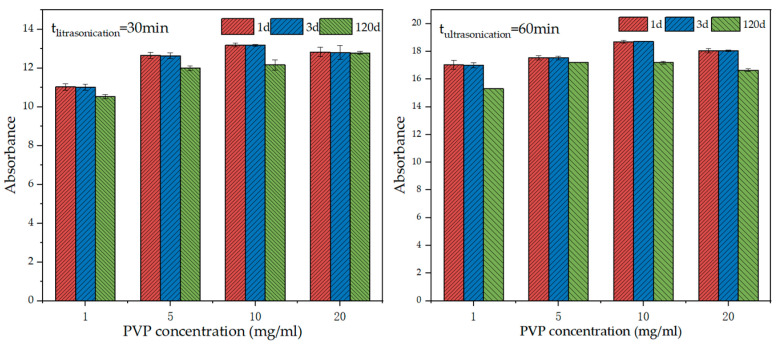
Absorbance of GNPs suspension at different PVP concentrations after various days of resting.

**Figure 4:**
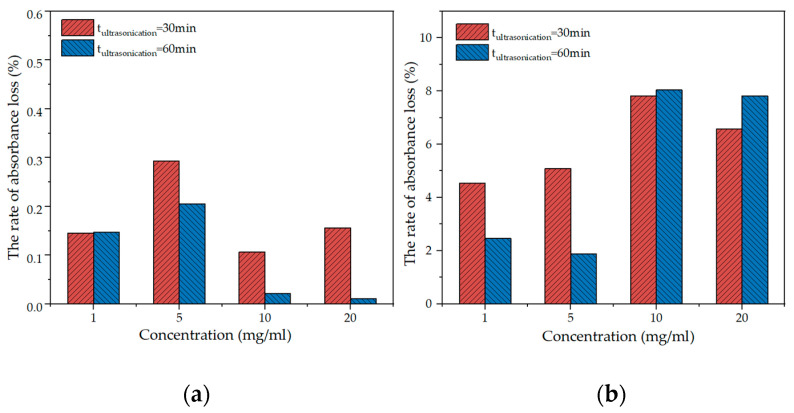
The rate of absorbance loss at different PVP concentrations after different days: (**a**) 3 d; (**b**) 120 d.

Figure 3 shows the absorbance of GNPs suspension at different concentrations after resting for 1 d, 3 d, and 120 days. No significant difference was observed after the 1 and 3 days of rest, and there was a mild decrease in absorbance up to 120 days. Figure 4 shows the rate of absorbance loss upon GNPs addition after 3 d and 120 days. When the PVP concentration of 10 mg/mL, the rate of absorbance loss was extraordinarily low. At 120 days, the absorbance of all GNPs suspensions at different PVP concentrations significantly decreased, particularly for the concentrations of 10 and 20 mg/mL. When the PVP concentration was 10 mg/mL, the rate of absorbance loss was 7.81% and 8.04% after 30 min and 60 min ultrasonication times, respectively. Such a low rate of absorbance loss indicates that the GNPs suspension of 10 mg/mL concentration had a good dispersion stability [42]. Meanwhile, there was no noticeable color change in GNPs suspensions at 3 and 120 days. Therefore, it may be concluded that the optimal PVP concentration is 10 mg/mL.

### 3.2. Optimization of Ultrasonication Time

**Figure 5:**
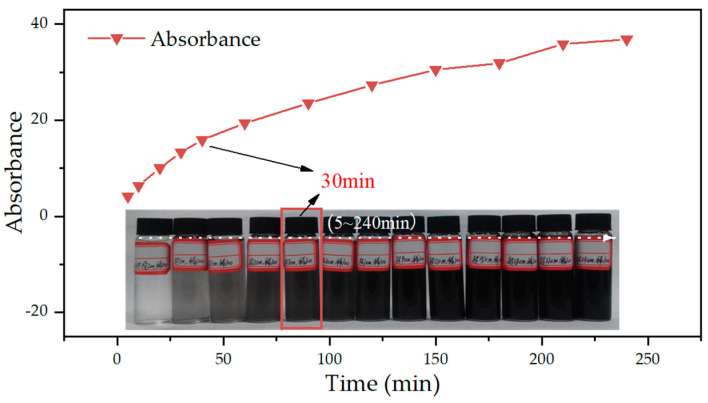
Absorbance of GNPs suspension in different ultrasonication time (inset: sample image).

Figure 5 shows the absorbance of GNPs in the aqueous solution with different ultrasonication times. With an increase in the ultrasonication time, the absorbance gradually increased. For example, the absorbance value increased by 32.69 from 5 min to 240 min. The reason was that the shear stress exerted by ultrasonication on the GNPs overcame the van der Waals forces between GNPs and thus improved their dispersion in the water [43,44,45]. The sample image (inset: Figure 5) shows that when ultrasonication is beyond 30 min, whereas the absorbance continues to increase, the color of each GNPs suspension has remained largely unchanged. The phenomena indicated that the electrostatic repulsive force existing between the internal tubular energy groups, such as hydroxyl groups, had not entirely overcome the van der Waals force between the GNPs [13], but enough to achieve dispersion significantly. Meanwhile, the existing studies found that too long ultrasonication time would disrupt the internal chain structure of GNPs, causing more severe GNPs agglomeration in cement paste [43,46]. Thus, 30 min was determined as the optimal ultrasonication time with the capability to preserve more of the GNPs structure.

### 3.3. Optimization of High-Speed Shear Time and Rate

**Figure 6:**
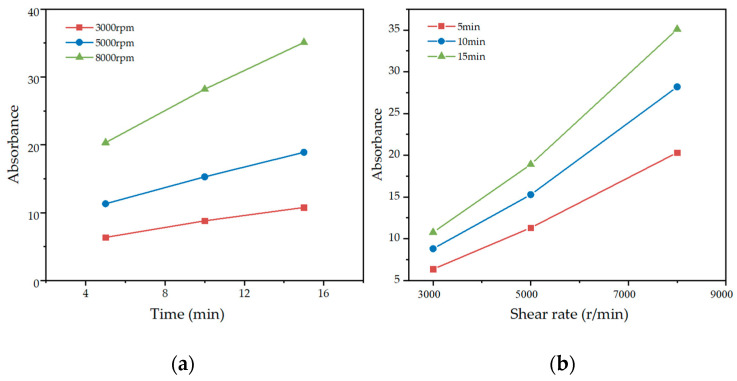
Absorbance of GNPs suspension; (**a**) different shear time; (**b**) different shear rates.

**Figure 7:**
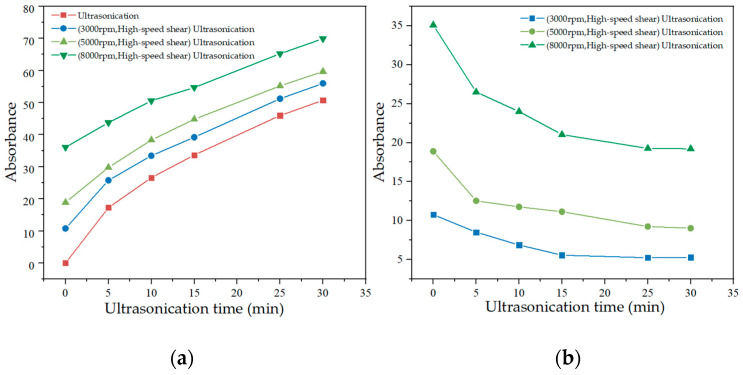
(**a**) A comparison between the absorbance of GNPs suspension under ultrasonication with and without high-speed shear; (**b**) The difference value between the absorbance of GNPs suspension under ultrasonication with and without high-speed shear.

**Figure 8:**
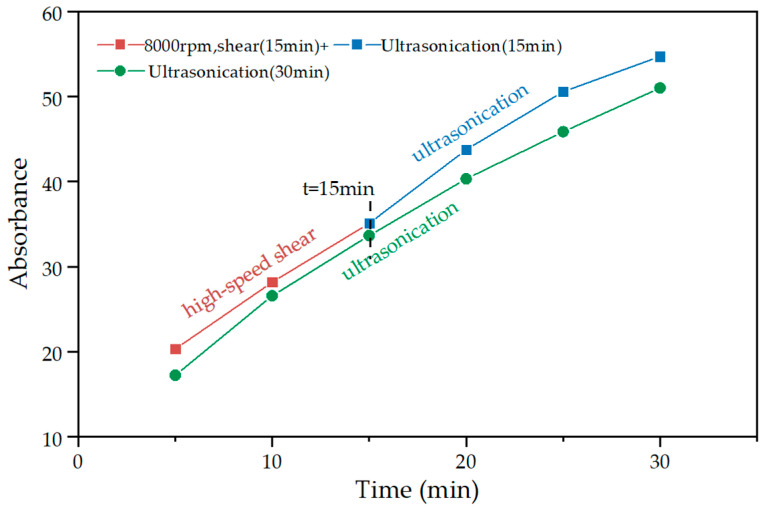
A comparison of high-speed shear (partial replacement ultrasonication) and only ultrasonication.

Figure 6 shows the absorbance of GNPs suspension at different high-speed shear times and rates. It can be seen that the absorbance of the suspension positively correlated to increased shear time and rate. The high-speed shear combined with ultrasonication was compared with ultrasonication only to determine the time of high-speed shear for ultrasonication replacement. Figure 7a shows that the absorbance of GNPs suspension under 30 min ultrasonication in combination with 15 min high-speed shear was higher than that of 30 min ultrasonication only. The absorbance of GNPs suspension after high-speed shear at 8000 rpm for 15 min was significantly larger than 15 min ultrasonication. Figure 7b shows the difference value of absorbance in GNPs suspension between the 30 min ultrasonication in combination with 15 min high-speed shear with different shear rates and 30 min ultrasonication only. It was noted that this difference value of absorbance became smaller as the ultrasonication time extension. When the GNPs suspension was treated by ultrasonication for more than 15 min, the difference value no longer changed obviously. Thus, 15 min high-speed shear at 8000 rpm was used to replace 15 min ultrasonication. Figure 8 reflects that the absorbance after the ultrasonication was replaced by high-speed shear was better than those using only ultrasonication. Thus, the first 15 min of the high-speed shear at 8000 rpm and the second 15 min of ultrasonication were to be determined. 

**Figure 9:**
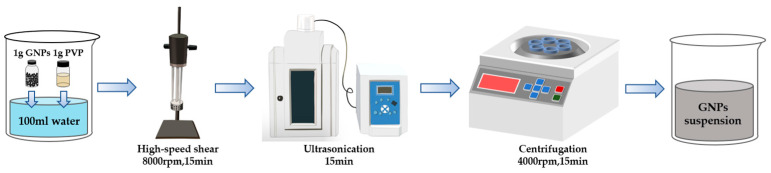
The optimal dispersion method of GNPs suspensions.

Figure 9 shows the optimal dispersion method to prepare GNPs suspensions.

### 3.4. Properties of GNPs Cement-Based Materials

#### 3.4.1. Setting Time

**Figure 10:**
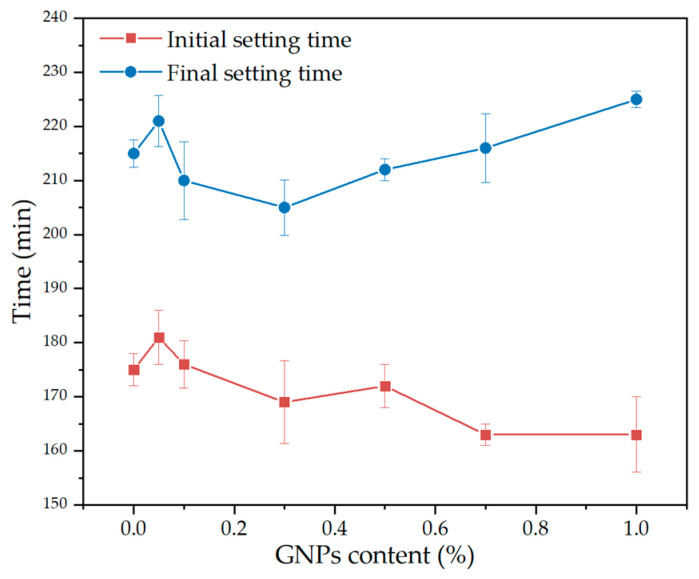
Influence of the dosage of GNPs on the setting time of cement paste.

Figure 10 shows the setting time of cement paste at various GNPs dosages. With the increase in GNPs dosage, the initial setting time tended to increase initially and then decrease. When the GNPs dosage was 1.0%, the initial setting time of cement paste decreased the most (163 min). This is because GNPs possess a large specific surface area, which could provide more nucleation sites during cement hydration and promote the early hydration of cement [13]. The final setting time increased to GNPs content of 0.05 wt% and then decreased. This could be attributed to the presence of a large number of functional groups (hydroxyl, hydroxy) on the surface of GNPs [41], which produced an electrostatic reaction in the alkaline environment and subsequently caused agglomeration and flocculation phenomenon [47]. The higher GNPs dosage also decreased the hydration degree of the cement paste [48]. However, such influence was not significant in the initial setting time. The differences in setting time are within 20 min compared with ordinary cement paste. Thus, all the mixing contents meet the use requirements.

#### 3.4.2. Flowability

**Figure 11:**
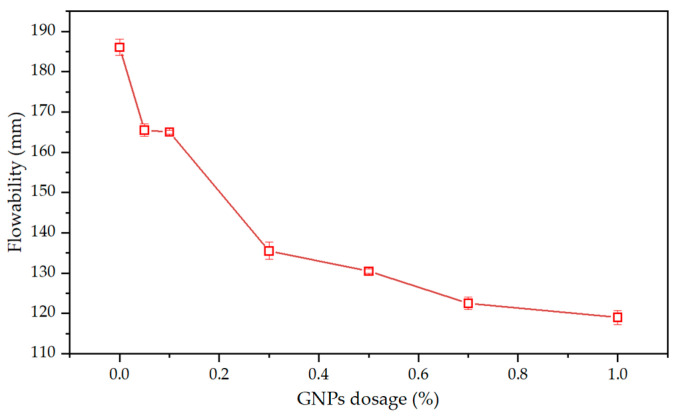
Influence of the GNPs dosage on the flowability of cement mortar.

As shown in Figure 11, the flowability decreased from 186 mm to 119 mm as the GNPs dosage increased. The reasons can be explained as follows: First, since the high specific surface area of GNPs could adsorb more water from the cement paste to its surface, resulting in a reduction of free water [41,49]. Second, when GNPs contacted with cement particles, the functional groups on its surface interacted electrostatically with those particles and subsequently produced the agglomeration and flocculation phenomenon [47]. This is due to the fact that the flocs sequestered a large amount of free water from the solution, which reduced the flowability of the fresh cement mortar [43]. Similar results have been demonstrated in other studies [43,50,51].

#### 3.4.3. Flexural and Compressive Strength 

**Figure 12:**
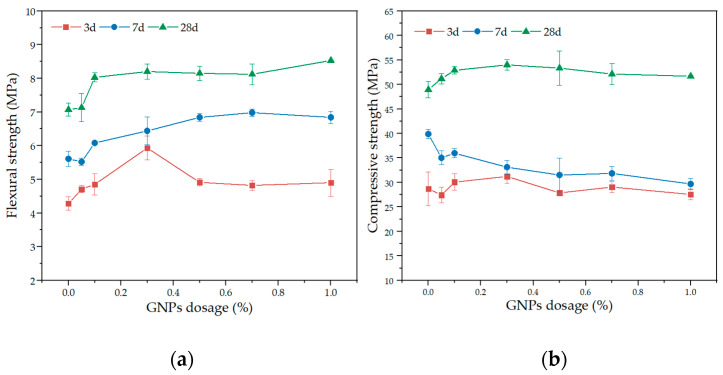
Mechanical properties of GNPs cement mortar; (**a**) flexural strength; (**b**) compressive strength.

Figure 12a,b shows the flexural and compressive strength of cement mortar with various GNPs dosages at 3 d, 7 d, and 28 d ages. At 3 d age, when the dosage of GNPs was less than 0.3 wt%, the flexural strength increased with the dosage increasing, while it decreased when the dosage of GNPs was greater than 0.3 wt%, which was similar to the compressive strength. At 7 d age, the higher GNPs dosage increased the flexural strength while it decreased the compressive strength. At 28 d age, both the flexural strength and compressive strength increased with GNPs dosage increasing. It should be noted that the 0.3 wt% GNPs dosage was optimal for strength gain. 

Some previous investigations found that the addition of dispersed GNP-based nanomaterials to cement mortar reduced the flexural and compressive strength of cement mortar since GNPs agglomerates formed weak areas in cement mortar, causing stress concentration [13,43]. Compared with the control group, the GNPs could improve the flexural and compressive strength of cement mortar, especially the early flexural strength. The underlying mechanism was related to the high surface area and wrinkled morphology of the GNPs, which increased the roughness of the interface between GNPs and the mortar matrix, thus enhancing the cohesive forces of the cement mortar [52,53]. However, when GNPs are over-added, agglomeration tends to occur in the cement matrix, which increases the porosity of the cement composite, thus adversely affecting the strength of the specimen [54]. It may be the combined role of both; the GNP additive in cement mortar hasn’t indicated significant mechanical properties improvement.

#### 3.4.4. Pressure-Sensitive Properties 

**Figure 13:**
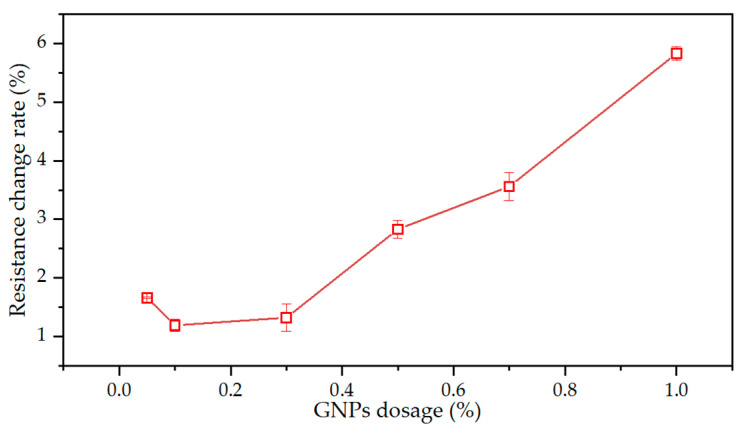
Pressure-sensitive property of GNPs cement mortar.

As shown in Figure 13, the rate of change in electrical resistance of GNPs first decreased and then increased slowly before 0.3 wt%, followed by a rapid increase from 0.3 wt% to 1.0 wt%. The rapid increase phenomenon was consistent with the percolation theory stage [55]. This theory referred to when the dosage of these GNPs in cement-based materials reached a critical value (0.3 wt%), and the conductivity increased abruptly. Furthermore, it should be noted that when the dosage of GNPs reached 1%, the rate of electrical resistance was 5.8%, which is enough to monitor structural health.

## 4. Conclusions

In this paper, we optimized the parameters of a combination dispersion method using PVP, ultrasonication, and high-speed shear for GNPs in an aqueous solution and investigated the effect of GNPs dosage on the mechanical and pressure-sensitive properties of cement-based materials. The following conclusions were drawn from the experimental results:

(1) An optimal dispersion method for GNPs in cement-based materials was developed, i.e., 10 mg/mL PVP addition, 15 min high-speed shear time at 8000 rpm, 15 min ultrasonication time, and 15 min centrifugation at 4000 rpm.
(2) The pressure-sensitive properties of cement mortar increased with GNPs dosage increasing. The cement mortar exhibited an optimal pressure sensitivity at 1% GNPs.
(3) The presence of GNPs promoted the hydration process and shortened the initial setting time of cement mortar. However, when the dosage of GNPs was high (0.7 wt%, 1.0 wt%), it would increase the final setting time. The flowability of the cement mortar was reduced with the increment of GNPs dosage.
(4) The incorporation of GNPs in cement mortar could improve their flexural and compressive strength. Moreover, when the GNPs dosage in cement mortar was 1.0%, it had the best effect on the improvement of flexural strength and later compressive strength.

## Figures and Tables

**Table 1:** Chemical compositions of cement and fly ash (wt%).

Components	SiO_2_	Al_2_O_3_	Fe_2_O_3_	CaO	Mgo	SO_3_
Cement	21.96	4.73	3.68	65.3	2.59	0.30
Fly ash	52.2	20.81	9.35	10.86	0.60	1.06

**Table 2:** Properties of GNPs (wt%).

Type	Specific Surface Area (m^2^/g)	Thickness (nm)	Mean Diameter (μm)	Bulk Density (g/cm^3^)
GNPs	150	6–8	15	0.07

**Table 3:** PVP Parameters.

K-Value	PH	N-Vinyl-2-Pyrrolidinpne	Water	Nitrogen Content	Formic Acid	Ignition Residue
27.0~32.4	3.0~5.0	≤0.001%	≤5.0%	11.5~12.8%	≤0.5%	≤0.1%

**Table 4:** Mix proportions of cement mortar.

Mix	GNPs (wt% of cement)	W/C	GNPs (kg/m^3^)	Cement (kg/m^3^)	Fly Ash (kg/m^3^)	Quartz Sand (kg/m^3^)	Water (kg/m^3^)	Thickener (kg/m^3^)	Superplasticizer (kg/m^3^)
Ref.	0	0.264	0	570.0	684.0	455.0	150.5	0.57	2.0
M-005	0.05	0.264	0.28	570.0	684.0	455.0	150.5	0.57	2.0
M-010	0.1	0.264	0.57	570.0	684.0	455.0	150.5	0.57	2.0
M-030	0.3	0.264	1.71	570.0	684.0	455.0	150.5	0.57	2.0
M-050	0.5	0.264	2.85	570.0	684.0	455.0	150.5	0.57	2.0
M-070	0.7	0.264	3.99	570.0	684.0	455.0	150.5	0.57	2.0
M-100	1.0	0.264	5.70	570.0	684.0	455.0	150.5	0.57	2.0

## Data Availability

The data presented in this study are available on request from the corresponding author.
